# Surgery

**Published:** 1877-05

**Authors:** 


### III. Surgery.

*A New Rectal Dilator and Explorer.* Ph. S. Wales. (New York Medical Rec., No. 329).

The treatment of stricture of the rectum is always tedious, painful and difficult, and in an increasing proportion to its distance from the anus. The malignant forms are uniformly fatal, and it is only in the non-malignant varieties that a successful issue may be secured by surgical measures. The ordinary appliances employed in the latter class of cases have not been altogether satisfactory, particularly in those instances in which the obstruction is seated high up in the bowel, and beyond the reach of the finger. Having recently had two cases of this latter sort, in one of which particularly the practical difficulties culminated from the height of the stricture, Dr. W. used a new kind of dilator with the most gratifying results. These dilators were manufactured out of pure rubber, with a canal running the whole length, and gradually increasing in size by an eighth, from a quarter of an inch to an inch in diameter. Each dilator is filled with a green sheath of corresponding dimensions, and if connected with a syringe or Politzer's air-bag, the sheath can be distended with water or inflated with air. The points of the dilators may terminate spherically or conically, the latter form, on the whole, being more easily introduced. The whole length of the sheath, both inside or outside the bowel, or any portion of it, may be filled with water; in the latter case a thread is to be twisted around the dilator at any point where it may be desirable to limit the distention.

"The method of introducing the dilator is simple. I prefer placing the patient, reclining on his left side, upon an ordinary operating-table, the thighs flexed and the buttocks just overhanging the lower edge. The operator may then comfortably seat himself during his manipulations—an advantage of no ordinary character, if they are to be at all prolonged. The smallest-sized instrument is smeared with grease, and its point inserted into the anus, and gently pushed onward in the following manner. The right hand grasps the dilator close to the anus, and the whole perineum is to be pressed upwards, which will advance the point of the instrument; the left hand now steadies it, while the right is slid downwards for a lower hold, the perineum of course settles with it; the dilator is again pushed forward in the same manner until the obstruction is passed. I have occasionally found that this may be greatly facilitated by sinking the fingers of the left hand deep into the left iliac region, and drawing upwards, as though an effort was being made, so to speak, to stretch out the sigmoid flexure, while pressure is maintained at the same time upon the dilator in the manner described. Another practical point of prime importance is to employ an abundant stream of water, projecting it through the conduit of the instrument as warm as can be comfortably borne, whenever its point is arrested from any cause. The water flowing from the distal aperture will distend the bowel, efface its folds, and break down any hardened fæces that may exist, obstructing the ascent of the dilator. While the operator is engaged with the dilator, an assistant may manage the syringe and throw in the water in such quantities as may be needed. It must be borne in mind, however, that no great volume should be used at once, otherwise the bowel will be excited to energetic contraction, and compel the dilator to be withdrawn before it has been properly lodged. In preliminary trials, the dilator may be permitted to remain two or three minutes, and afterwards, when greater tolerance is established, a longer stay may be allowed. I rarely exceed half an hour in any case, even when the patient makes no complaint of irritation or pain. After several introductions of one size of the dilators, perhaps seven or eight, the next largest may be taken, and so on until the stricture has been sufficiently expanded. The application of the instrument may be repeated twice or thrice a week, according to circumstances, such as the irritability of the rectum, temperament of the individual, and intercurrent attacks of diarrhœa, or other trouble. Twice a week, in my experience, suffices in most cases of rectal catheterism. A fortunate issue, if attainable, can only be brought about by patient and prolonged treatment. Rudeness or violence, inflicted with a view of hastening the case, can effect nothing but harm, and may jeopardize the life of the patient. If the instruments be hastily thrust in, the bowels may be perforated, especially in those cases in which inflammatory softening or ulceration exists; or, if they be too large, the rectal mucous membrane may be ruptured, giving rise to smart hemorrhage, as happened in one of my early cases; or the entire wall of the bowel may be ruptured into the peritoneum—an accident that is pretty sure to be followed by peritonitis, with all of its attendant dangers.

"These funest sequences are infinitely less liable to follow the use of pure india-rubber dilators than that of any other sort; for certainly, *a priori*, nothing could furnish a milder, more equable, and less dangerous force than these, and experience sustains this view.

"I have also been using the dilators in applying ointments, variously medicated with astringent and anodyne substances, in cases of relaxation of the intestinal mucous membrane, prolapse, hemorrhoidal tumors, and other morbid rectal conditions. A slight modification converts the dilator into an irrigator; that is, by having two parallel conduits in it—one for the fluid to pass in, and the other for it to escape externally. The bowel may be thus irrigated quite high up, for I have frequently inserted the instrument twenty-eight inches into the colon. In one case the patient passed every morning one or two large, yeasty stools, mixed with mucus, and followed by much mental depression. A cure was obtained by the injection, three times a week, of a solution of carbolic acid, after internal medication had failed. Much advantage may be realized from the use of warm irrigation in various diseases of the contiguous pelvic and abdominal organs—as cystitis, prostatitis, suppression of urine, and intussusception. The half-inch dilator will furnish a ready tube for introducing alimentary substances into the stomach or rectum.

"A reliable explorer for diagnosing intestinal diseases may be prepared in the following manner: take a dilator, say one-half an inch in diameter, and over its distal extremity draw a hood of thin india rubber two inches long, securing its margin with a fine silken thread. If the rubber is not at hand, a piece of moistened bladder or gold beater's-skin will answer very well. The instrument thus made ready should be well coated with a stiff grease, like simple cerate, or zinc ointment, which works better than sweet oil in facilitating its gliding over the mucous membrane, and introduced in the manner described for the dilator, until its point is lodged far above in the descending colon. A syringe is attached to the instrument, and its point expanded into the form of a ball an inch or more in diameter. Gentle traction is now to be made, which will cause the ball filled with water to move slowly down the bowel. If no obstacle is present, the ball will soon emerge from the anus; on the contrary, any obstruction will arrest it above. It may be that spasmodic contraction will have a like effect, but this can be easily distinguished from permanent stricture by keeping up the traction a few moments, until the muscular force of the intestinal walls is exhausted, when the ball will again slip along. In cases of organic narrowing, its degree may be determined approximately by maintaining the traction on the explorer, while the water is permitted to flow out in small quantities at a time, until the size of the ball is sufficiently reduced to slip past the constriction; the diameter of the ball, which represents that of the stricture, may be now ascertained by mere inspection.

*Shortening of the Lower Limb after Fracture of the Femur.* WRIGHT. (*Archives of Clinical Surgery*, February, 1877).

A very rational way of explaining the reason of shortening of the lower limb after fracture, is by making comparative measurements of lower limbs that never have been broken. Dr. W. has accomplished this, by a careful measurement of the lower limbs of sixty cases; giving the age, occupation, and nativity of each. The maximum disparity in length, was one and three-eighths of an inch; the minimum, one-eighth of an inch. All these measurements were made in the usual way, from the most prominent point of the anterior superior spine of the ileum, to the lower extremity of the internal malleolus. After a careful, and lengthy investigation of lower limb shortening, the author arrives at the following conclusions:

"First. The lower limbs of the same person are not always of the same length.

Secondly. The greater number of lower limbs, comparing the limbs of the same person, show a difference in length.

Thirdly. The normal lower limbs that I have measured, give the fol lowing result: The left lower limb is oftener longer than the right, and the right lower limb is nearly as often longer than the left.

Fourthly. About one person out of every five has lower limbs that measure the same length.

Fifthly. I have measured the lower limbs of cadavers and of skeletons, the soft parts having been removed by dissection. These measurements confirm the above results."

The cause of normal lower limb shortening, he says, may be deficient development of the epiphyseal cartilage, that is, one of the cartilages might continue to grow, and ossify after the other had completed its developments; or "a variation in the length and obliquity of the neck of the femur, incident to the age of the patient, may not occur during the same time, and with equal pace in the two femoral necks." A mistake might be easily made in a case, where there was equal length in the normal lower limbs, by the superior spines of the ileum occupying comparatively different positions; one spine projecting a little further down, or a little further up, than its fellow; or one of the malleoli might extend downwards a little further than the one of the opposite side. The author believes that it is impossible to say just how much a broken femur had shortened a limb; the only correct and reliable way would be, to measure the limb before and after the injury, knowing that such a marked differerence exists in the length of normal limbs, it would seem unscientific, and even perilous to apply extension and counter extension so forcibly, as to make an injured limb, where there is considerable shortening, as long as the other. The author says, that in some cases of fracture of the femur, he has easily made the broken limb as long as the other, and supposed it was chiefly due to skillful treatment; while in other cases, equal, and even more care would be exercised, yet the patient would get up with an inch shortening.

W. F. L.

*A New and Simple Operation for the Removal of Laryngeal Polypi.* Prof. Voltolini. (*Monatsschrift f. Ohrenheilk. and Halskrankh*, 1877. No. 2.)

This new operation which scarcely can be honored with this name, is nothing more or less than the removal of soft polypi in the larynx by a swabbing method. The only instrument required is a piece of soft sponge securely fastened to flexible wire of German silver. The sponge must not be over one centimetre broad; else it cannot be introduced into the larynx. Where the presence of a soft or pendulus polypus has been ascertained by the larynx mirror, the growth is removed without the aid of the mirror, in this manner:

"The patient pulls out his tongue, and the surgeon depressing forcibly the root of the latter with a spatula brings the epiglottis in view. As soon as the epiglottis can be seen, the sponge (which was previously softened in warm water) is passed into the throat, until it reaches the posterior surface of the epiglottis on which it is made to glide down into the larynx. As soon as the sponge has entered the larynx, it is grasped firmly by a spasmodic closure of the latter; and the whole knack of my method consists in this, *that while the larynx is thus closed, no attempt is made at pushing the sponge further down*; *it should be left in its position until the larynx opens again*, which it must do very soon, because the patient wants to breathe. At the instant when this occurs *i. e.* when the glottis is opened, the sponge is passed down through it. This act is very easy for the patient, as it were, inhales the sponge. Once under the glottis the sponge is moved up and down to swab the vocal cords, and to tear off the polypous growth. If the polypus sits on the upper surface of the vocal cords, it is of course unnecessary for the sponge to get through the glottis; it is then sufficient that the probang has entered the larynx and is turned around in it while the glottis is contracted, for just then the sponge is sure not to miss the proliferous growth." Six cases are reported in the above article that have been treated by this method. V. thinks his operation is applicable to the soft polypi only; but for these which constitute the majority of growths in the larynx, it will supersede all other operations, because it can be performed at once without any preliminary preparations and without the aid of the laryngoscope; because every practitioner can do it by ordinary daylight, and because it will obviate the necessity of tracheotomy or laryngotomy in cases of imminent suffocation due to so excessive a proliferation of soft polypi that they fill the entire lumen of the larynx.

